# The clinical efficacy of a daratumumab-based regimen in relapsed/refractory acute leukemia: a single-center experience

**DOI:** 10.1007/s00277-024-05892-9

**Published:** 2024-07-24

**Authors:** Yi Dai, Lin Luo, Zhenbin Wei, Peng Cheng, Jun Luo, Jing Li

**Affiliations:** Department of Hematology, The First Affiliated Hospital of Guangxi Medical University, Nanning, Guangxi 530021 China

**Keywords:** Relapsed/refractory acute leukemia, Daratumumab-based salvage regimen, CD38, Venetoclax

## Abstract

Relapsed/refractory acute leukemia (R/R-AL) is associated with a low remission rate, short survival rate, and poor prognosis. Treating R/R-AL remains challenging as there is no standardized effective regimen; hence, there is a need for efficient therapies. CD38 expression has been observed in acute myeloid leukemia (AML) and acute lymphoblastic leukemia (ALL). Daratumumab is a humanized anti-CD38 monoclonal antibody used to treat multiple myeloma and has been reported to treat R/R-AL safely and effectively. The clinical data of 10 adult patients with R/R-AL who were treated with a daratumumab-based salvage regimen between July 2018 and May 2023 at our center were analyzed retrospectively. Seven AML and three ALL cases were included in the analysis. Seven (70%) patients showed responses to the treatments (complete response [CR], 60%; partial response [PR], 10%). Of the seven responders, three underwent allogenic stem cell transplantation (ASCT), including one who underwent a second ASCT. Among the five patients with R/R AML who had prior exposure to venetoclax, three achieved a therapeutic response (two CR and one PR) when re-treated with venetoclax in combination with daratumumab. The median follow-up time was 6.15 months (0.9–21 months). Overall survival and event-free survival rates at 12 months were 68.6% and 40.0%, respectively. The main adverse events included grade 3 febrile neutropenia (20%) and grade 3 hematological toxicities (60%). The daratumumab-based salvage regimen offers patients with R/R-AL the opportunity of remission with acceptable tolerability, creating the possibility of bridging ASCT.

**Supplementary Information:**

The online version contains supplementary material available at 10.1007/s00277-024-05892-9.

## Introduction

Acute leukemia (AL) is a highly heterogeneous, malignant, hematological tumor with a high recurrence rate. AL is primarily classified as acute myeloid leukemia (AML) and acute lymphoblastic leukemia (ALL). The 2-year overall survival (OS) rates for relapsed and refractory cases are 27% and 29%, respectively [1–3]. Allogeneic stem cell transplantation (ASCT) is the sole therapeutic option to ensure long-term disease-free survival in a refractory case or following a relapse. However, this strategy is limited because few patients who receive salvage therapy achieve complete remission (CR). Therefore, it is important to develop a salvage regimen that can serve as an effective bridging treatment between relapse/refractory disease and transplantation [1].

CD38, a 45-kDa single-chain transmembrane glycoprotein receptor with bifunctional ectoenzymatic activity, is present in several hematological malignancies [4]. On the other hand, normal lymphoid and myeloid cells as well as several non-hematopoietic organs express CD38 at very low levels [4]; making it an ideal target for AL. Daratumumab, a United States Food and Drug Administration-approved monoclonal antibody, targets a specific CD38 epitope and is well tolerated and efficacious in multiple myeloma (MM) [5]. Preclinical data has recently shown the efficacy of daratumumab in AL [6], and several case reports have demonstrated its clinical efficacy in patients with the condition [7–9]. Here, we present a retrospective observational study of patients with relapsed/refractory acute leukemia (R/R-AL) who received a daratumumab-based salvage regimen; findings from the study will provide real-world data on the safety and efficacy of this drug.

## Materials and methods

### Methods

10 cases of R/R-AL were retrospectively analyzed in adult patients (age ≥ 18 years) treated with a daratumumab-based salvage regimen from July 2018 to May 2023 in our center. The objective response rate (ORR), CR, event-free survival, OS, and adverse events (AEs) in this cohort were assessed. Univariate analysis was also used to attempt to analyze the factors affecting the efficacy of the salvage therapy. The study design was approved by the institutional review committee (Approval No: 2024-E299-01), and all investigations were conducted in accordance with the Declaration of Helsinki.

### Relevant definitions

Centralized laboratories measured minimal residual disease (MRD) using flow cytometry (FCM) or real-time quantitative reverse transcriptase polymerase chain reaction (RQ-PCR). MRD negative (MRD-) was defined as FCM detection or RQ-PCR < 0.01%. MRD positive (MRD+) was defined as FCM detection or RQ-PCR ≥ 0.01%. Complete remission (CR) was characterized by the absence of extramedullary lesions, a bone marrow (BM) blast cell count < 5%, an absolute neutrophil count ≥ 1 × 10^9^/L, and a platelet count ≥ 100 × 10^9^/L. Complete remission with incomplete recovery (CRi) was defined by an absolute neutrophil count < 1 × 10^9^/L and a platelet count < 100 × 10^9^/L, with other criteria mirroring those for CR. Partial remission (PR) involved a reduction in BM blast cell count to 5–20%. Non-remission (NR) was defined as BM blasts > 20%. Progressive disease (PD) was associated with either extramedullary disease or a 25% increase in peripheral blood or BM blasts. Disease relapse was defined as the reemergence of blast cells in peripheral blood or BM (≥ 5%) or extramedullary disease in patients who had achieved CR. The overall response rate (ORR) was the proportion of patients achieving CR, CRi, or PR. Overall survival (OS) was calculated from the first day of salvage treatment until death or the last follow-up. Event-free survival (EFS) commenced from the start of salvage therapy to recurrence, death, or last follow-up. Adverse events (AEs) were characterized and evaluated using the National Cancer Institute Common Terminology Criteria for Adverse Events, version 5.0.

### Statistical analysis

Differences among categorical variables were compared using Fisher’s exact test or the chi-squared test. The Kaplan–Meier method was used to estimate the probability of OS. The comparison between baseline variables and response was explored using Fisher’s exact test, chi-squared test, Mann–Whitney U test, and t-test, as appropriate. Statistical significance was set at *P* < 0.05. The cut-off date for this analysis was May 1st, 2023, and data were analyzed using SPSS 26.0 statistical software.

## Results

### Patient characteristics

The 10 R/R-AL cases, four males and six females, included seven AML cases, one B-ALL case, and two T-ALL cases. The median patient age was 39 years. Three patients (3/10, 30%) were in salvage line 1, six patients (6/10, 60%) in salvage line 2, and one patient (1/10,10%) in salvage line 3. The median line of prior treatments was two. Of the 10 patients, five had refractory leukemia, while the other five had relapsed leukemia (one of the patients with AML relapsed after ASCT). Patient characteristics are summarized in Table 1.

**Table 1:** Patients’ characteristics

Number	Sex	Age	Disease	Types	Prior therapies line	Genetic findings	Cytogenetics
1	M	18	AML^a^	Refractory	3	WT-1, RUNX1, FLT3-TKD	46,X, del(Y)
2	F	47	AML	Relapsed	1	DupMLL, DNMT3A, IDH1	Normal karyotype
3	F	67	AML	Relapsed	2	-	Complex karyotype
4	F	40	AML	Refractory	2	NRAS, BCOR	Normal karyotype
5	M	44	AML	Relapsed	1	WT1, NRAS, IDH	t (2,6) (q37;p21.3)
6	F	33	AML	Refractory	2	SF3B1	Complex karyotype
7	F	37	AML	Relapsed	1	MYC, PHF6, TTN, TET2, WT1	+ 8
8	F	38	B-ALL^b^	Refractory	2	MLL, EVT1, IKZF1, *BCR::ABL (p190)*	t(9;22) (q34;q11)
9	M	25	T-ALL^c^	Refractory	2	WT1, TP53, JAK3	Normal karyotype
10	M	52	T-ALL	Relapsed	2	*SIL::TAL-1*	-8

^a^Acute Myeloid Leukemia, ^b^B-cell Acute Lymphoblastic Leukemia, ^c^T-cell Acute Lymphoblastic Leukemia

### Treatment administration

Adult patients with R/R-AL were administered a daratumumab-based regimen (16 mg/kg); however, AML patients are mainly treated with a demethylating agent and venetoclax. Different treatment schemes for patients with ALL are highlighted in Supplementary Table S1.

### Treatment efficacy

**Fig. 1:**
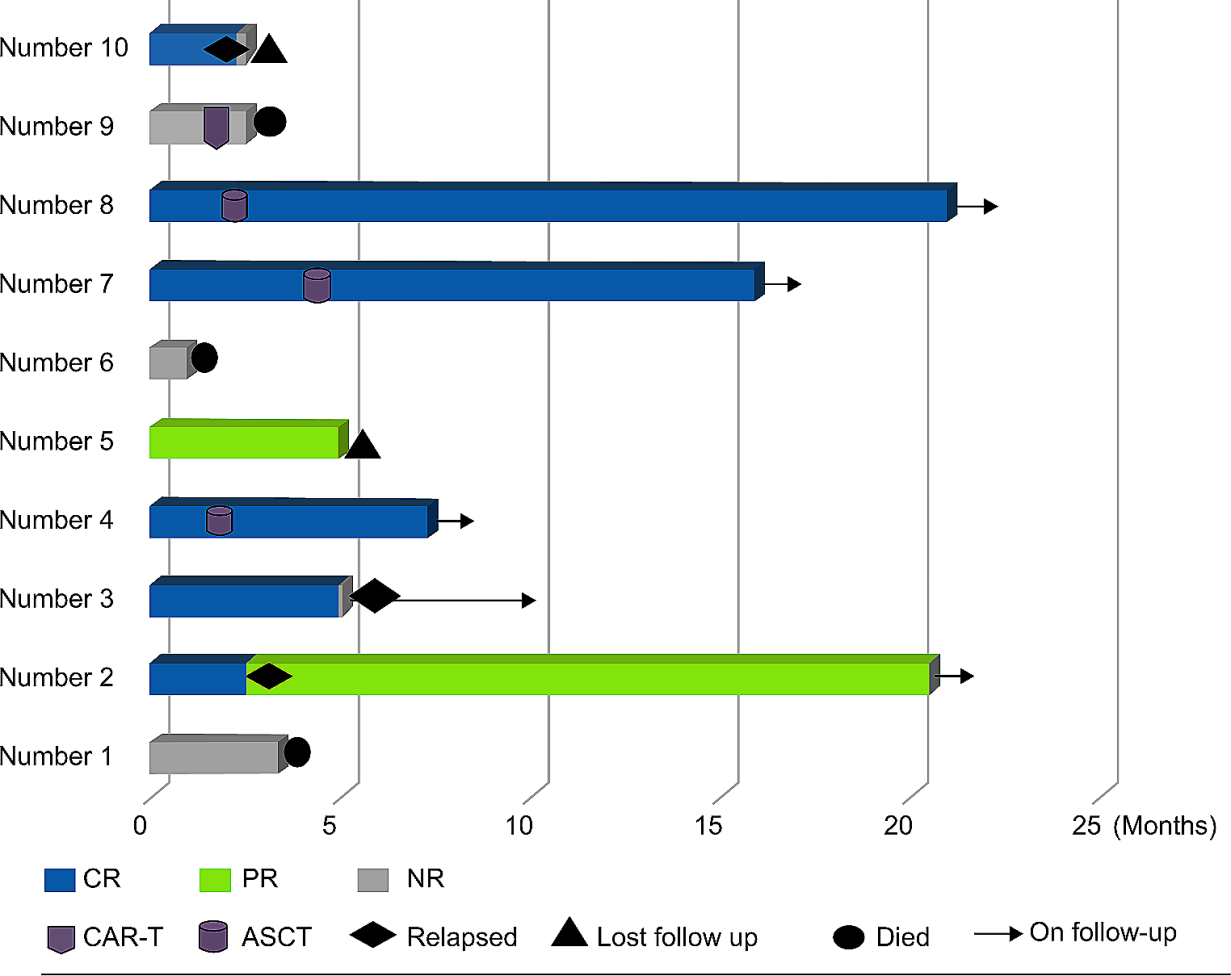
Swim plot. *CR* Complete remission, *PR* Partial remission, *NR* Non-remission, *CAR-T* Chimeric antigen receptor T-cell therapy, *ASCT* Allogeneic stem cell transplantation

Ten patients were treated with one to four doses of daratumumab, with a median dose number of two. Six patients achieved CR, and one patient achieved PR. The ORR and CR + CRi rates were 70% and 60%, respectively. Of the responders, three patients (two AML cases and one B-ALL case) received ASCT, and two patients (two AML cases) received consolidation chemotherapy (the swim plot is shown in Fig. 1).

**Fig. 2:**
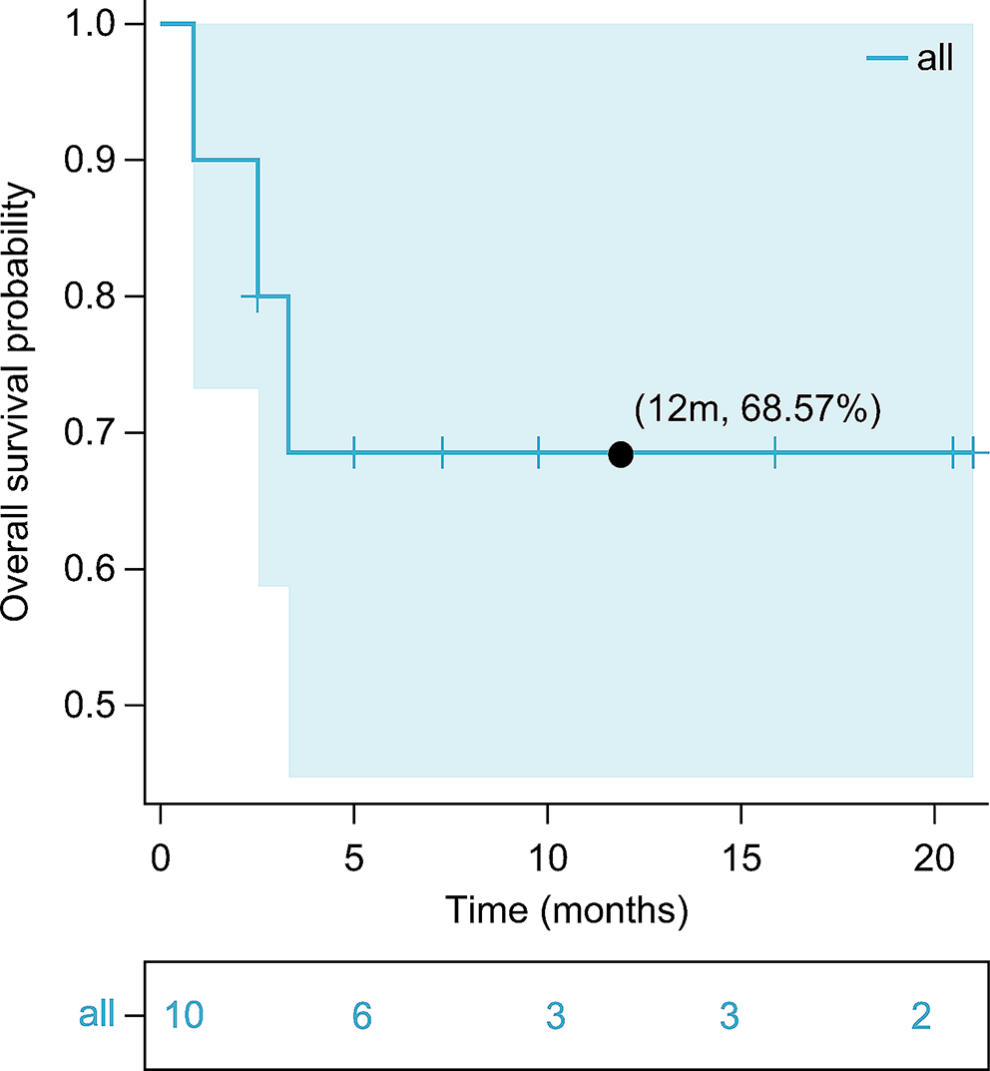
Twenty-one-month overall survival

**Fig. 3:**
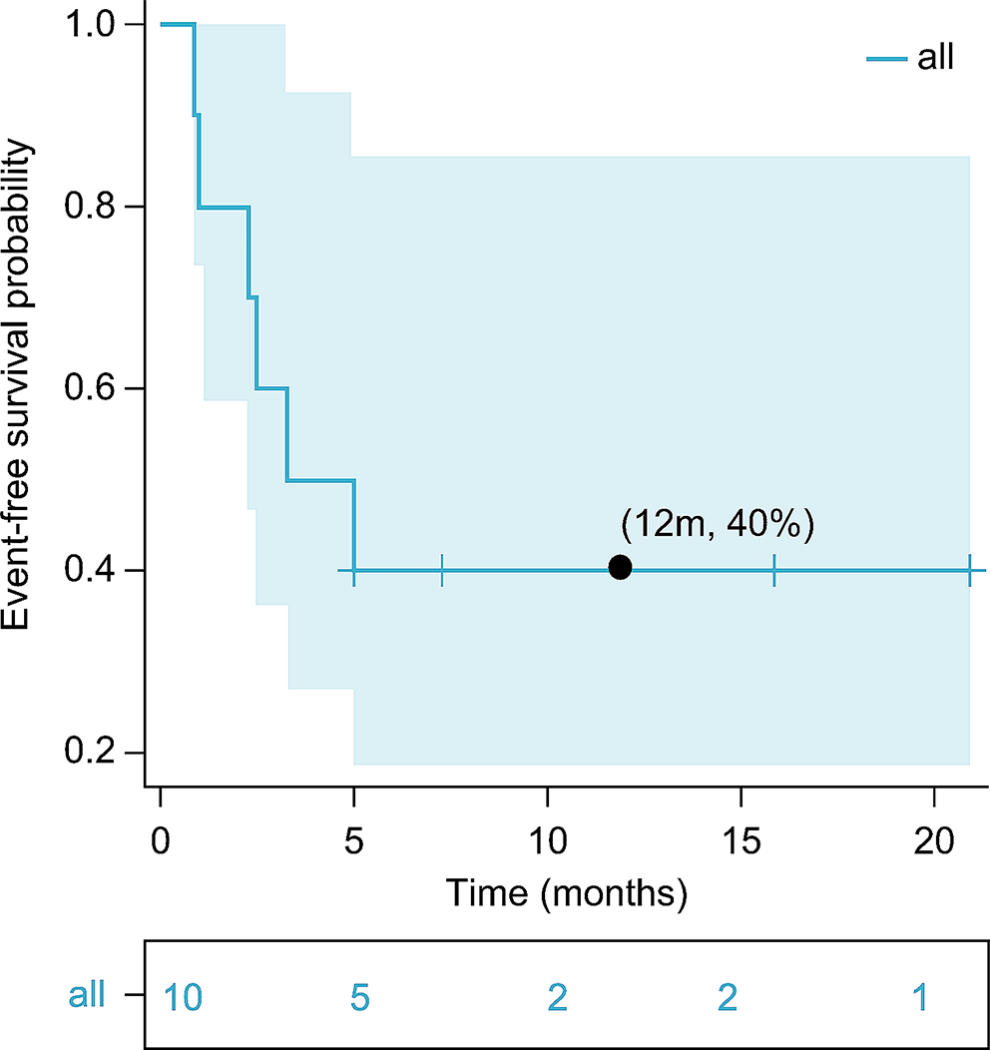
Twelve-month event-free survival

Among the five patients with R/R AML who had prior exposure to venetoclax, three achieved a therapeutic response (two CR and one PR) when re-treated with venetoclax in combination with the daratumumab regimen. Changes in leukemia burden and CD38 expression levels before and after treatment are shown in Table 2. With a median follow-up time of 6.15 months (range, 0.9–21 months), the OS and EFS rates at 12 months were 68.6% and 40.0%, respectively (Figs. 2 and 3).

**Table 2:** Leukemia burden of patients before and after treatment

Number	Before treatment			After treatment			Status
	BM^a^ blasts %	FCM^b^ %	CD38%	BM %	FCM %	CD38%	
1	70	20.1	100	54.5	65.35	45.50	NR^c^
2	19	10.91	85.4	4.5	< 0.001	0	CR^d^
3	20	1.8	94.7	1.3	< 0.001	0	CR
4	5	0.3	99.7	2.5	< 0.001	0	CR
5	8	1.46	98.9	7	-	-	PR^e^
6	76	35.54	52.3	30	-	-	NR
7	13	1.42	96.86	3.5	< 0.001	0	CR
8	15	4.26	91.14	2	< 0.001	0	CR
9	10	6.27	90.58	69	20.31	-	NR
10	29.5	10.00	96.23	0.8	< 0.001	0	CR

^a^Bone marrow, ^b^Flow cytometry, ^c^Non-remission, ^d^Complete remission, ^e^Partial remission

The possible variables associated with this response were subsequently investigated. No statistically significant differences were observed between responders and non-responders in terms of sex (*P* = 0.50), type of disease (*P* = 1.0), previous lines of treatment (*P* = 0.101), or BM blasts (*P* = 0.221). Finally, the potential correlation between the degree of CD38 expression and the response was assessed. CD38 expression levels were not significantly different in terms of response or non-response (94.56 ± 4.92% vs. 76.15 ± 33.73%, *P* = 0.581) (Table 3).

**Table 3:** Predictors of response to daratumumab

Variable	Responders (*N* = 7)	Non-responders (*N* = 3)	*P*
Sex			0.50
Male	2	2	
Female	5	1	
Type of disease			1.0
AML^a^	5	2	
ALL^b^	2	1	
Previous lines of treatment			0.101
1	3	0	
2	4	2	
3	0	1	
BM^c^ blasts, %	15.64 ± 8.18	52.17 ± 36.21	0.221
CD38, %	94.56 ± 4.92	76.15 ± 33.73	0.581

^a^Acute Myeloid Leukemia, ^b^Acute lymphoblastic leukemia, ^c^Bone marrow

### AEs in daratumumab-based regimen-treated patients

Regarding the AEs associated with the daratumumab-based regimen (Table 4), a temporary infusion reaction was noted in 10% of the patients. Hematological AEs, including leukopenia, anemia, thrombocytopenia, neutropenia, febrile neutropenia, and hypokalemia of any grade, were observed in 70%, 10%, 70%, 80%, 30%, and 60% of patients, respectively. Leukopenia, anemia, thrombocytopenia, neutropenia, and febrile neutropenia of grade 3 or higher occurred in 60%, 40%, 50%, 50%, and 20% of patients, respectively. No induction-related death was observed. The tolerability of the daratumumab regimen was acceptable in the context of R/R-AL.

**Table 4:** Adverse events

Toxicity	ALL^a^ Grade, *N* (%)	Grade 3/4, *N* (%)
Leukopenia	7 (70)	6 (60)
Anemia	10 (10)	4 (40)
Thrombocytopenia	7 (70)	5 (50)
Neutropenia	9 (90)	5 (50)
Hypokalemia	6 (60)	1 (10)

^a^Acute lymphoblastic leukemia

## Discussion

Treatment of R/R-AL remains a major challenge. More than half of all patients who are newly diagnosed eventually fall into the R/R category and face a bleak prognosis. ASCT shows promise for long-term survival. However, because the pre-transplant leukemia load affects transplant outcomes, leukemia-directed therapy should first achieve a disease response. Even in cases where ASCT is not an option, OS is linked to leukemia-directed treatment efficacy. Thus, effective second-line treatment is crucial [10, 11]. While emerging targeted immunotherapies such as gemtuzumab, blinatumomab, inotuzumab, ozogamicin, and CAR-T cells have significantly improved the outcomes for R/R-AL, the approved treatment options for R/R-AL remain unsatisfactory [12].

CD38 is a 45-kDa single-chain transmembrane glycoprotein with intricate biological characteristics. It has enzymatic activity that can regulate Ca^2+^ flow, affect multiple intracellular signaling pathways, and act as a receptor or adhesion factor to regulate cell proliferation, apoptosis, and other metabolic activities [13–15].

Daratumumab, an IgG1j human monoclonal antibody (mAb), binds to a unique CD38 epitope. It has a broad-spectrum killing activity. Daratumumab binding to CD38 on tumor cells induces complement-dependent cytotoxicity, antibody-dependent cellular cytotoxicity and phagocytosis, tumor cell apoptosis, and CD38 enzymatic activity regulation [4]. Furthermore, daratumumab has been tested against various hematological cancers after performing well in MM treatment without significant toxicity [16].

Daratumumab showed significant efficacy in suppressing the proliferation of ALL and AML tumors with elevated CD38 expression in preclinical studies [6]. Daratumumab is believed to have multiple mechanisms of action, including blocking the transfer of mitochondria from BM mesenchymal stem cells to leukemic blasts. Additionally, it improves the tumor microenvironment, allowing for immune evasion. These mechanisms can be considered additional ways in which this drug works in AML treatment [17, 18]. Following preliminary data, a few reports have demonstrated the efficacy of daratumumab in patients with advanced ALL and without other therapeutic options [19]. However, there have been few reports on the therapeutic efficacy of daratumumab in AML. This study focused on targeting CD38 in patients with R/R-AL (AML and ALL). We retrospectively analyzed the clinical data of 10 patients with R/R-AL (seven AML and three ALL) treated with daratumumab. In our study, most patients were heavily pre-treated and had a high disease burden.

For relapsed/refractory (R/R) ALL with standard chemotherapy induction, CR rates were 30–46% in the first salvage setting and 18–25% in the second salvage setting [11, 20–22]. CR rates in salvage settings ranged from 12 to 75% with the use of venetoclax-based regimens [23–25].

In our study, all three R/R ALL cases followed second-line treatment. Two patients received treatment in combination with chemotherapy, while one was treated with a venetoclax-based regimen. The CR rate achieved was 66.7%. Notably, there appears to be a trend towards improved efficacy compared to data previously reported. Case 1, having previously been exposed to vincristine and dasatinib, experienced remission after these drugs were combined with daratumumab. Therefore, based on these observations of clinical efficacy, we hypothesize that daratumumab may exhibit a unique antitumor effect in certain R/R ALL patients. However, due to the retrospective nature of the study and its small sample size, it is challenging to determine the specific role of daratumumab when used in combination with chemotherapy or a venetoclax-based regimen.

For R/R AML, commonly used salvage chemotherapy regimens for younger patients include FLAG-IDA (fludarabine, cytarabine, idarubicin, and granulocytecolony-stimulating factor) and MEC (mitoxantrone, etoposide, and cytarabine), with reported CR rates ranging from 40 to 65% [26]. Responses to salvage chemotherapy for older adults are notably lower, at only 30% [26]. In our study of seven R/R AML cases, the CR rate was 57.1%, which is comparable to historical data. However, among the five R/R AML patients previously exposed to venetoclax, three achieved a therapeutic response—two CRs and one PR—when re-treated with venetoclax in combination with the daratumumab regimen. Preclinical models suggest that venetoclax may exhibit synergistic effects with daratumumab [16]. Therefore, we hypothesize that this combination could potentially enhance efficacy in R/R AML, although further data are required to support this.

We measured CD38 expression before and after daratumumab treatment. Nine patients had high expression levels, ranging from 85.4 to 100%, whereas one patient had a low expression level of 52.3%. Patients with low CD38 expression did not respond to treatment. However, univariate analysis did not show a relationship between CD38 expression level and efficacy, which may be due to the limitations of retrospective studies and small sample sizes. Patients who responded to the treatment showed a significant decrease in CD38 levels. Whether AL with low or negative CD38 expression can benefit from daratumumab treatment warrants further investigation as this antibody not only targets cancer directly but also promotes an antitumor immune response [15, 27]. Additionally, the mean BM blast percentage in daratumumab responders was numerically lower than that of non-responders (15.6% versus 52.17%), although this difference was not statistically significant due to small sample sizes.

Safety assessments showed that the main AEs included grade 3 febrile neutropenia (20%) and grade 3 hematological toxicities (60%). Daratumumab-based regimens were well tolerated by the patients.

The main limitations of this study are the heterogeneity of the patient population (AML + ALL) and of the treatment received, as well as the small number of cases.

Our study found that in certain R/R ALs, patients achieved efficacy even though Dara had been included in previously administered regimens. This suggests that Dara exerts specific anti-leukemic effects. It is worthwhile to investigate how to identify R/R ALs where Dara will be truly beneficial.

## Electronic supplementary material

Below is the link to the electronic supplementary material.

Supplementary Material 1

## Data Availability

The datasets used and/or analysed during the current study are available from the corresponding author on reasonable request.

## References

[1] Forman SJ, Rowe JM (2013) The myth of the second remission of acute leukemia in the adult. Blood 121(7):1077–1082. 10.1182/blood-2012-08-234492 23243288 10.1182/blood-2012-08-234492 PMC3575753

[2] Breems DA, Van Putten WL, Huijgens PC et al (2005) Prognostic index for adult patients with acute myeloid leukemia in first relapse. J Clin Oncol 23(9):1969–1978. 10.1200/JCO.2005.06.027 15632409 10.1200/JCO.2005.06.027

[3] Ganzel C, Sun Z, Cripe LD et al (2018) Very poor long-term survival in past and more recent studies for relapsed AML patients: the ECOG-ACRIN experience. Am J Hematol 93(8):1074–1081. 10.1002/ajh.25162 29905379 10.1002/ajh.25162 PMC6699929

[4] Van De Donk NW, Janmaat ML, Mutis T et al (2016) Monoclonal antibodies targeting CD38 in hematological malignancies and beyond. Immunol Rev 270(1):95–112. 10.1111/imr.12389 26864107 10.1111/imr.12389 PMC4755228

[5] Van De Donk NW, Moreau P, Plesner T et al (2016) Clinical efficacy and management of monoclonal antibodies targeting CD38 and SLAMF7 in multiple myeloma. Blood 127(6):681–695. 10.1182/blood-2015-10-646810 26631114 10.1182/blood-2015-10-646810

[6] Naik J, Themeli M, De Jong-Korlaar R et al (2019) CD38 as a therapeutic target for adult acute myeloid leukemia and T-cell acute lymphoblastic leukemia. Haematologica 104(3):e100–e103. 10.3324/haematol.2018.192757 30190344 10.3324/haematol.2018.192757 PMC6395314

[7] Zhang Y, Xue S, Liu F, Wang J (2020) Daratumumab for quick and sustained remission in post-transplant relapsed/refractory acute lymphoblastic leukemia. Leuk Res 91:106332. 10.1016/j.leukres.2020.106332 32126433 10.1016/j.leukres.2020.106332

[8] Mirgh S, Ahmed R, Agrawal N et al (2019) Will daratumumab be the next game changer in early thymic precursor-acute lymphoblastic leukaemia? Br J Haematol 187(2):e33–e35. 10.1111/bjh.16154 31452197 10.1111/bjh.16154

[9] Stanulla M, Schewe DM, Bornhauser B et al (2023) Molecular complete remission following combination treatment of daratumumab and venetoclax in an adolescent with relapsed mixed phenotype acute leukemia. Ann Hematol 102(3):669–672. 10.1007/s00277-023-05083-y 36651980 10.1007/s00277-023-05083-y PMC9977701

[10] Sedov V, Stuart RK (2017) Vosaroxin in relapsed/refractory acute myeloid leukemia: efficacy and safety in the context of the current treatment landscape. Ther Adv Hematol 8(6):185–195. 10.1177/2040620717703012 28567238 10.1177/2040620717703012 PMC5424861

[11] Fielding AK, Richards SM, Chopra R et al (2007) Outcome of 609 adults after relapse of acute lymphoblastic leukemia (ALL); an MRC UKALL12/ECOG 2993 study. Blood 109(3):944–950. 10.1182/blood-2006-05-018192 17032921 10.1182/blood-2006-05-018192

[12] Norsworthy KJ, Ko CW, Lee JE et al (2018) FDA approval summary: Mylotarg for treatment of patients with relapsed or refractory CD33-positive acute myeloid leukemia. Oncologist 23(9):1103–1108. 10.1634/theoncologist.2017-0604 29650683 10.1634/theoncologist.2017-0604 PMC6192608

[13] Mehta K, Shahid U, Malavasi F (1996) Human CD38, a cell-surface protein with multiple functions. FASEB J 10(12):1408–1417. 10.1096/fasebj.10.12.8903511 8903511 10.1096/fasebj.10.12.8903511

[14] Deaglio S, Mehta K, Malavasi F (2001) Human CD38: A (r)evolutionary story of enzymes and receptors. Leuk Res 25(1):1–12. 10.1016/s0145-2126(00)00093-x 11137554 10.1016/s0145-2126(00)00093-x

[15] Konopleva M, Estrov Z, Zhao S, Andreeff M, Mehta K (1998) Ligation of cell surface CD38 protein with agonistic monoclonal antibody induces a cell growth signal in myeloid leukemia cells. J Immunol 161(9):4702–4708. 10.4049/jimmunol.161.9.4702 9794400

[16] Zhong X, Ma H (2022) Targeting CD38 for acute leukemia. Front Oncol 12:1007783. 10.3389/fonc.2022.1007783 36313735 10.3389/fonc.2022.1007783 PMC9597453

[17] Mistry JJ, Moore JA, Kumar P et al (2021) Daratumumab inhibits acute myeloid leukaemia metabolic capacity by blocking mitochondrial transfer from mesenchymal stromal cells. Haematologica 106(2):589–592. 10.3324/haematol.2019.242974 32193250 10.3324/haematol.2019.242974 PMC7849566

[18] Patiño-Escobar B, Ramos R, Linares M, Mejía A, Alcalá S (2020) CD38: from positive to negative expression after daratumumab treatment. Cureus 12(4):e7627. 10.7759/cureus.7627 32399359 10.7759/cureus.7627 PMC7213648

[19] Cerrano M, Bonifacio M, Olivi M et al (2022) Daratumumab with or without chemotherapy in relapsed and refractory acute lymphoblastic leukemia. A retrospective observational campus ALL study. Haematologica 107(4):996–999. 10.3324/haematol.2021.279851 35021604 10.3324/haematol.2021.279851 PMC8968887

[20] Gökbuget N, Stanze D, Beck J et al (2012) Outcome of relapsed adult lymphoblastic leukemia depends on response to salvage chemotherapy, prognostic factors, and performance of stem cell transplantation. Blood 120(10):2032–2041. 10.1182/blood-2011-12-399287 22493293 10.1182/blood-2011-12-399287

[21] O’Brien S, Thomas D, Ravandi F et al (2008) Outcome of adults with acute lymphocytic leukemia after second salvage therapy. Cancer 113(11):3186–3191. 10.1002/cncr.23919 18846563 10.1002/cncr.23919 PMC4188532

[22] Tavernier E, Boiron JM, Huguet F et al (2007) Outcome of treatment after first relapse in adults with acute lymphoblastic leukemia initially treated by the LALA-94 trial. Leukemia 21(9):1907–1914. 10.1038/sj.leu.2404824 17611565 10.1038/sj.leu.2404824

[23] Goursaud L, Berthon C, Quesnel B (2023) Successful bridging to cell therapy for relapsed/refractory acute lymphoblastic leukaemia with a combination of venetoclax and PEG-asparaginase. Br J Haematol 200(4):e37–e39. 10.1111/bjh.18595 36470305 10.1111/bjh.18595

[24] Pullarkat VA, Lacayo NJ, Jabbour E et al (2021) Venetoclax and Navitoclax in combination with chemotherapy in patients with relapsed or refractory acute lymphoblastic leukemia and lymphoblastic lymphoma. Cancer Discov 11(6):1440–1453. 10.1158/2159-8290.CD-20-1465 33593877 10.1158/2159-8290.CD-20-1465 PMC9533326

[25] Gibson A, Trabal A, Mccall D et al (2021) Venetoclax for children and adolescents with acute lymphoblastic leukemia and lymphoblastic lymphoma. Cancers 14(1):150. 10.3390/cancers14010150 35008312 10.3390/cancers14010150 PMC8750927

[26] Rashidi A, Weisdorf DJ, Bejanyan N (2018) Treatment of relapsed/refractory acute myeloid leukaemia in adults. Br J Haematol 181(1):27–37. 10.1111/bjh.15077 29318584 10.1111/bjh.15077

[27] Cerrano M, Castella B, Lia G et al (2020) Immunomodulatory and clinical effects of daratumumab in T-cell acute lymphoblastic leukaemia. Br J Haematol 191(1):e28–e32. 10.1111/bjh.16960 32686081 10.1111/bjh.16960

