# Supplementary material for: The clinical efficacy of a daratumumab-based regimen in relapsed/refractory acute leukemia: a single-center experience
Source: Ann Hematol. 2024 Jul 24;103(10):4057–63. doi: 10.1007/s00277-024-05892-9 (PMC11512853; doi:10.1007/s00277-024-05892-9)
Supplement: Supplementary file 1 — Supplementary Material 1 [file 277_2024_5892_MOESM1_ESM.docx]

**Supplementary Information**

*Annals of Hematology*

**The clinical efficacy of a daratumumab-based regimen in relapsed/refractory acute leukemia: A single-center experience**

Yi Dai^1^, Lin Luo^1^, Zhenbin Wei^1^, Peng Cheng^1^, Jun Luo^1^, Jing Li^1^*

^1^Department of Hematology, the First Affiliated Hospital of Guangxi Medical University, Nanning, Guangxi 530021, China

***Corresponding author:** Jing Li

Email: [nikky97@163.com](mailto:nikky97@163.com)

**Supplementary Table S1** Daratumumab chemotherapy regimens for patients with R/R-AL

| Number | Type of Chemotherapy |
| --- | --- |
| 1 | DARA+DAC+VEN |
| 2 | DARA+AZA+VEN |
| 3 | DARA+AZA+VEN |
| 4 | DARA+HHT+AZA+VEN |
| 5 | DARA+AZA+VEN |
| 6 | DARA+AZA+VEN |
| 7 | DARA+AZA+VEN |
| 8 | DARA+VCR+Dasatinib |
| 9 | DARA+DAC+VEN |
| 10 | DARA+ Hyper CVAD-B+PEG-Asp |

R/R-AL, Relapsed/refractory acute leukemia; DARA, daratumumab; DAC, decitabine; VEN, venetoclax; AZA, azacitidine; VCR, vincristine; HHT, homoharringtonin; PEG-Asp, pegaspargase
